# Comparative Noise Analysis of Readout Circuit in Hemispherical Resonator Gyroscope

**DOI:** 10.3390/mi16070802

**Published:** 2025-07-09

**Authors:** Zhihao Yu, Libin Zeng, Changda Xing, Lituo Shang, Xiuyue Yan, Jingyu Li

**Affiliations:** 1 College of Advanced Interdisciplinary Studies, National University of Defense Technology, Changsha 410073, China; yuzhihao@hrbeu.edu.cn (Z.Y.); zenglibin19@nudt.edu.cn (L.Z.); xing.changda@foxmail.com (C.X.); s323520082@hrbeu.edu.cn (L.S.); yanxiuyue24@163.com (X.Y.); 2 College of Intelligent Systems Science and Engineering, Harbin Engineering University, Harbin 150001, China

**Keywords:** Hemispherical Resonator Gyroscope (HRG), Transimpedance Amplifier (TIA), Charge-Sensitive Amplifier (CSA), noise characteristics, circuit bandwidth

## Abstract

In high-precision Hemispherical Resonator Gyroscope (HRG) control systems, readout circuit noise critically determines resonator displacement detection precision. Addressing noise issues, this paper compares the noise characteristics and contribution mechanisms of the Transimpedance Amplifier (TIA) and Charge-Sensitive Amplifier (CSA). By establishing a noise model and analyzing circuit bandwidth, the dominant role of feedback resistor thermal noise in the TIA is revealed. These analyses further demonstrate the significant suppression of high-frequency noise by the CSA capacitive feedback network. Simulation and experimental results demonstrate that the measured noise of the TIA and CSA is consistent with the theoretical model. The TIA output noise is 25.8 $\mu V_{\mathrm{rms}}$, with feedback resistor thermal noise accounting for 99.8%, while CSA output noise is reduced to 13.2 $\mu V_{\mathrm{rms}}$, a reduction of 48.8%. Near resonant frequency, the equivalent displacement noise of the CSA is $1.69\times 10^{-14}\;m/\sqrt{\mathrm{Hz}}$, a reduction of 86.7% compared to the TIA’s $1.27\times 10^{-13}\;m/\sqrt{\mathrm{Hz}}$, indicating the CSA is more suitable for high-precision applications. This research provides theoretical guidance and technical references for the topological selection and parameter design of HRG readout circuits.

## 1. Introduction

The Hemispherical Resonator Gyroscope (HRG) is a critical component in high-precision positioning and navigation systems, holding significant importance in strategic emerging industries such as high-end equipment manufacturing, aerospace, and intelligent transportation. The HRG, introduced in the 1960s, utilizes the vibrational mode changes within a hemispherical resonator to detect external angular velocity, simplifying the mechanical structure and enhancing reliability and lifespan [1]. Compared to traditional mechanical and optical gyroscopes, the HRG offers high precision, reliability, radiation resistance, extended lifespan, and strong environmental adaptability, making it suitable for applications in spacecraft, industrial robots, autonomous driving, and unmanned underwater vehicles [2,3].

HRGs typically utilize a uniformly distributed electrode structure to detect resonator vibrational modes [4]. The current signals collected by each electrode correspond to the orthogonal components of the resonator’s vibrational modes, which are then vectorially combined to accurately reconstruct the resonator’s mode shape information. In this process, the readout circuit converts the weak detection current signals into processable voltage signals [5]. The low amplitude of these detection signals makes them highly susceptible to circuit noise, which in turn affects the detection accuracy and stability of the gyroscope control system.

Current mainstream readout circuit schemes include Transimpedance Amplifier (TIA), Charge-Sensitive Amplifier (CSA), and switched-capacitor structures [6,7,8]. Among these, the Capacitance-to-Voltage (C-V) conversion circuit based on the TIA is the most widely used due to its simple structure and fast response. A T-shaped resistor network-based transimpedance amplifier was reported in 2007 [9]. It achieved a high transimpedance gain of 25 MΩ in an integrated circuit with a power consumption of 400 μW, and a measured baseline noise of $0.02\;\mathrm{aF}/\sqrt{\mathrm{Hz}}$. Research indicates that the T-shaped structure’s noise performance is significantly degraded compared to conventional TIAs under the same equivalent impedance [10]. Notably, the CSA scheme proposed by the University of Freiburg, Germany, effectively suppresses the noise of traditional transimpedance amplifiers by using a feedback capacitor instead of a resistor. However, an analysis of the noise suppression mechanism is lacking. Experimental data shows that this circuit achieves a dynamic range of 92 dB within a 40 Hz bandwidth, with a full-scale range exceeding ±1400°/s [11]. Recently, Yinyu Liu analyzed the noise in TIA circuits and found that the thermal noise introduced by the feedback resistor is the primary noise source, while the readout circuit based on the CSA effectively suppresses the resistor thermal noise [12]. However, it should be noted that the noise model established in this study does not fully consider the impact of circuit bandwidth on noise characteristics, and subsequent research needs to further improve the matching between the theoretical model and experimental verification.

This paper addresses the noise characteristics of the HRG readout circuit. First, the HRG detection mechanism is analyzed, along with the operational principles of TIA and CSA topologies. Then, a noise model is established in conjunction with circuit bandwidth, and the noise characteristics and contribution mechanisms of TIA and CSA are comparatively analyzed. Finally, the validity of the noise model and characteristics analysis is confirmed via simulation and experimental circuit testing.

## 2. HRG Detection Principle

### 2.1. Dynamic Model of the HRG

**Figure 1:**
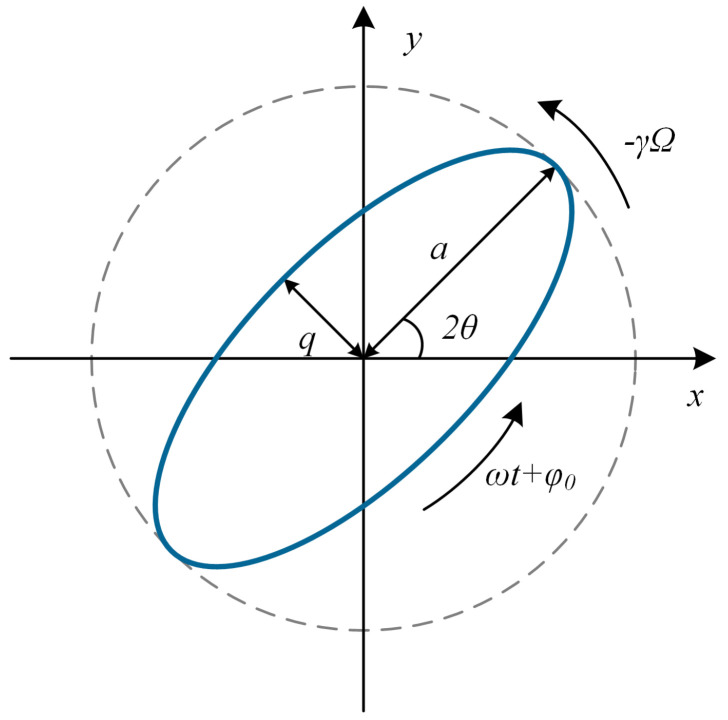
Schematics illustrating the trajectory of oscillation in an HRG under open-loop operation.

Ideally, the motion trajectory of the HRG in Cartesian coordinates can be modeled as a two-degree-of-freedom vibration system, as shown in Figure 1.

As shown above, *a* denotes the major axis of the elliptical orbit, corresponding to the principal wave amplitude, and *q* represents the minor axis, corresponding to the quadrature wave amplitude, respectively. θ indicates the angle between the principal wave amplitude and the 0° electrode axis. $\varphi_{0}$ denotes the initial phase angle of the standing wave pattern. ω represents the resonant frequency of the resonator. The corresponding dynamic equation is given by (1).

(1) $$\left[\begin{array}{c} \ddot{x}\\ \ddot{y} \end{array}\right]+\frac{2}{\tau }\left[\begin{array}{c} \dot{x}\\ \dot{y} \end{array}\right]+\omega^{2}\left[\begin{array}{c} x\\ y \end{array}\right]=\frac{1}{m}\left[\begin{array}{c} F_{x}\\ F_{y} \end{array}\right]+\left[\begin{array}{cc} 0 & 4\gamma \Omega \\ -4\gamma \Omega & 0 \end{array}\right]\left[\begin{array}{c} \dot{x}\\ \dot{y} \end{array}\right],$$

where *x* and *y* denote vibration displacements along two mutually orthogonal axes in the Cartesian coordinate system. $F_{x}$ and $F_{y}$ represent electrostatic forces applied to the respective axes. Ω indicates the input angular velocity. γ is the precession factor of the resonator [13].

The general solution to (1) is given by (2).

(2) $$\left\{\begin{array}{c} x=a\cos2\theta \cos\left(\omega t+\varphi_{0}\right)-q\sin2\theta \sin\left(\omega t+\varphi_{0}\right)\\ y=a\sin2\theta \cos\left(\omega t+\varphi_{0}\right)+q\cos2\theta \sin\left(\omega t+\varphi_{0}\right), \end{array}\right.$$

where $\theta =\theta_{0}-\gamma \int \Omega dt$.

### 2.2. Detection Equation of the HRG

**Figure 2:**
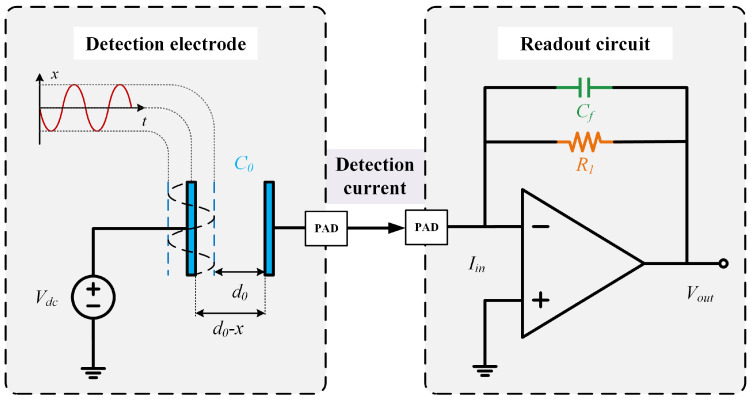
Schematic of HRG detection, illustrating the operation of the detection electrode and readout circuit. The detection current from the detection electrode is conducted via the PAD and wires to the readout circuit, yielding the detection signal required for subsequent processing.

The HRG detection section comprises detection electrodes and a readout circuit. The detection electrodes utilize a parallel-plate capacitor structure to detect capacitance variations induced by resonator vibration [14]. The readout circuit converts the minute detection current generated by the capacitance change into a voltage signal via components in the feedback network, as illustrated in Figure 2.

The plate displacement *x* characterizes the resonator amplitude, while $d_{0}$ denotes the initial plate spacing. $V_{dc}$ is the amplitude of the DC excitation signal. ∆C represents the change in detection capacitance, expressed as (3). Under high-voltage conditions, changes in the plate spacing of the parallel-plate capacitor alter the capacitance value. To maintain voltage stability, the charge quantity must be dynamically adjusted, thereby generating a detection current $I_{in}$, expressed as (4).

(3) $$∆C=\frac{\varepsilon S}{d_{0}-x},$$

(4) $$I_{in}=\frac{d∆Q}{dt}=V_{dc}\frac{d∆C}{dt},$$

where ε is the vacuum permittivity, and *S* is the area of the parallel-plate electrodes.

### 2.3. Readout Circuit Topologies and Operating Principle

**Figure 3:**
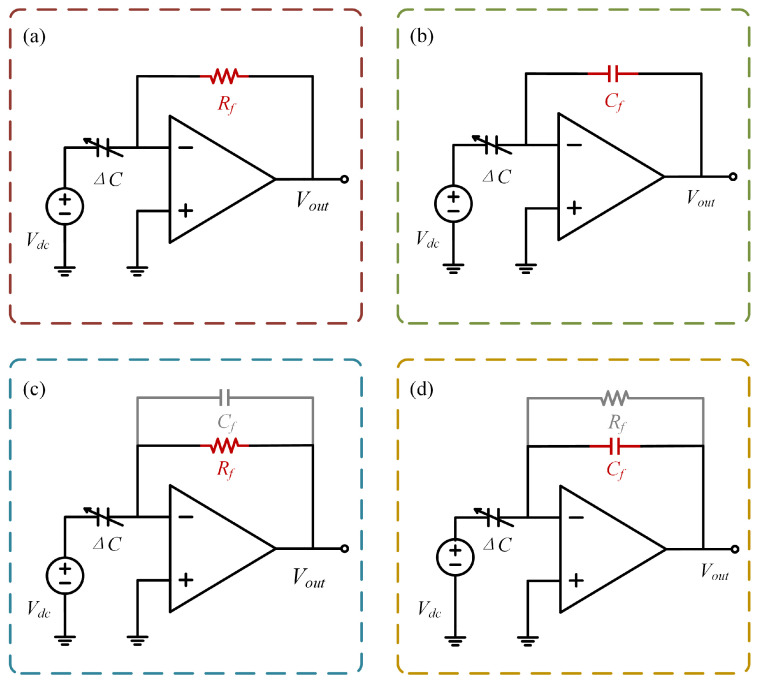
Readout circuit topologies. (**a**) Ideal TIA, (**b**) ideal CSA, (**c**) practical TIA with RC compensation, (**d**) practical CSA with anti-saturation design.

This paper focuses on the TIA and CSA within the readout circuit, deriving the detection equations for both topologies. As shown in Figure 3, although the TIA and CSA share similar topologies, their operating principles differ fundamentally. Theoretically, the ideal TIA converts the detection current signal into a voltage output via a feedback resistor. The CSA, however, utilizes a feedback capacitor as an integrating element, achieving C-V conversion through charge accumulation [15].

The fundamental operational principles of the ideal TIA and CSA define their input–output behavior as described below.

(5) $$V_{TIA}=-R_{f}I_{in},$$

(6) $$V_{CSA}=-\frac{1}{C_{f}}\int I_{in}dt.$$

Substituting (3) and (4) into (5) and (6) yields (7) and (8), respectively.

(7) $$V_{TIA}=-R_{f}V_{dc}\frac{d}{dx}\left(\frac{\varepsilon S}{d_{0}-x}\right)\frac{dx}{dt}=-R_{f}V_{dc}\frac{\varepsilon S}{d_{0}^{2}}{\left(1-\frac{x}{d_{0}}\right)}^{-2}\frac{dx}{dt},$$

(8) $$V_{CSA}=-\frac{V_{dc}}{C_{f}}\int \frac{d}{dx}\left(\frac{\varepsilon S}{d_{0}-x}\right)dx=-\frac{V_{dc}}{C_{f}}\frac{\varepsilon S}{d_{0}^{2}}\int {\left(1-\frac{x}{d_{0}}\right)}^{-2}dx.$$

After performing Taylor expansions on (7) and (8) while neglecting higher-order terms in *x*, we simplify the detection equations for the TIA and CSA schemes to the forms given in (9) and (10), respectively.

(9) $$V_{TIA}=-R_{f}V_{dc}\frac{C_{0}}{d_{0}}\frac{dx}{dt}\left[1+2\frac{x}{d_{0}}+3{\left(\frac{x}{d_{0}}\right)}^{2}+4{\left(\frac{x}{d_{0}}\right)}^{3}\right]\approx -R_{f}V_{dc}\frac{C_{0}}{d_{0}}\frac{dx}{dt},$$

(10) $$V_{CSA}=-\frac{V_{dc}}{C_{f}}\frac{C_{0}}{d_{0}}\int [1+2\frac{x}{d_{0}}+3{\left(\frac{x}{d_{0}}\right)}^{2}+4{\left(\frac{x}{d_{0}}\right)}^{3}]dx\approx -\frac{V_{dc}}{C_{f}}\frac{C_{0}}{d_{0}}x.$$

where $C_{0}$ represents the initial capacitance value of the parallel-plate capacitor, i.e., the detection capacitance value at x=0. ω represents the frequency of the input current, which is identical to the gyroscope’s resonant frequency.

In engineering practice, both circuits require structural optimization. For the TIA, a compensation capacitor is typically connected in parallel with the feedback resistor, forming an RC low-pass filter whose cutoff frequency is determined by $f_{c}=1/2\pi R_{f}\;C_{f}$, effectively suppressing high-frequency interference signals [16]. For the CSA, due to the integrator’s infinite gain for DC bias signals, output saturation is prone to occur [17]. Therefore, a high-value resistor is connected in parallel with the feedback capacitor, forming dual paths of high-frequency integration and low-frequency discharge, thereby maintaining the circuit’s operational stability. The improved input–output relationship of the readout circuit in the frequency domain is as follows:

(11) $$V_{out}\left(s\right)=-\frac{V_{dc}∆CR_{f}s}{1+R_{f}C_{f}s}.$$

**Figure 4:**
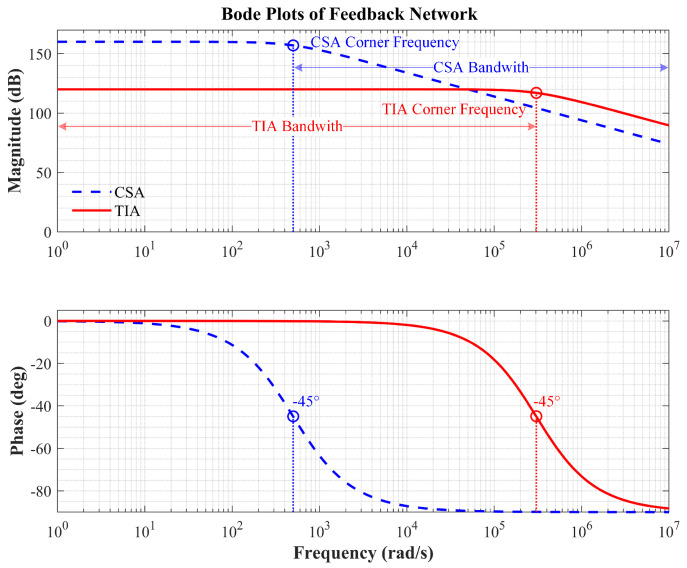
Bode plots of feedback network, including magnitude response curves and phase response curves.

Bode plot analysis reveals significant differences in the frequency-domain characteristics of the TIA and CSA, as illustrated in Figure 4. The TIA primarily operates in the low-frequency band, with its operating bandwidth determined by the cutoff frequency of the RC low-pass filter. The CSA, on the other hand, exhibits high-frequency response characteristics, with its effective operating bandwidth limited by the signal bandwidth.

Based on the circuit characteristics, (11) can be simplified, resulting in:

(12) $$V_{out}\left(s\right)=\left\{\begin{array}{cc} -V_{dc}∆CR_{f}s & TIA\;(R_{f}\ll \frac{1}{C_{f}s}),\\ -\frac{V_{dc}∆C}{C_{f}} & CSA\;(R_{f}\gg \frac{1}{C_{f}s}). \end{array}\right.$$

The operating mode of the readout circuit depends on the dominance of capacitance and resistance in the RC feedback network. When the resistance impedance in the feedback network is much greater than the capacitance, the circuit behaves as a TIA. Conversely, it behaves as a CSA. Let s=jω, and perform a comparative analysis of the gain and phase characteristics of both circuits. The results indicate that the TIA circuit gain is affected by the resonant frequency, while the CSA does not exhibit this issue. Furthermore, the phase difference of the output signals between the two circuits is 90°.

## 3. Readout Circuit Noise Characteristics Analysis

### 3.1. Noise Model Construction and Characteristics Analysis

**Figure 5:**
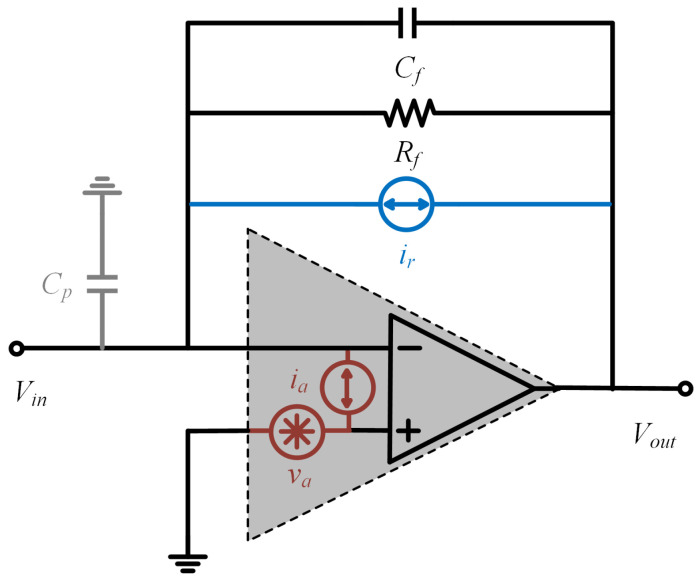
Noise model for readout circuits based on three primary noise sources.

A detailed investigation of readout circuit noise is essential for high-precision readout circuit design. The primary contributors to readout circuit noise are the thermal noise of resistors, the input voltage noise of the operational amplifier, and the input current noise of the operational amplifier. The following analysis constructs a readout circuit noise model based on these three noise sources and analyzes their characteristics, as shown in Figure 5.

Advancements in semiconductor manufacturing processes have significantly reduced the flicker noise components in the input voltage and current noise of high-precision, low-noise operational amplifier chips. Within a typical operating frequency range, the voltage and current noise can be simplified to white noise for analysis.

#### 3.1.1. Operational Amplifier Input Voltage Noise

The operational amplifier input voltage noise is $v_{a}$. The influence of parasitic capacitance in the circuit must be considered during the analysis. According to Kirchhoff’s Current Law [18]:

(13) $$\frac{v_{a}}{\frac{1}{C_{p}s}}=\frac{e_{v}-v_{a}}{\frac{R_{f}}{1+R_{f}C_{f}s}}.$$

Further calculation yields the voltage noise transfer function, denoted as ${NTF}_{v}$, which defines the relationship between the input voltage noise and output voltage noise in the operational amplifier.

(14) $${NTF}_{v}=\frac{1+R_{f}\left(C_{p}+C_{f}\right)s}{1+R_{f}C_{f}s}.$$

The output noise power spectral density (PSD) corresponding to the input voltage noise is given by (15). Integrating this output noise yields the mean-square noise voltage, as expressed in (16).

(15) $$e_{v}^{2}={\left|{NTF}_{v}\right|}^{2}v_{a}^{2}.$$

(16) $$V_{n,v}^{2}=\frac{v_{a}^{2}}{2\pi R_{f}C_{f}}\left[\alpha +\left(2\frac{C_{p}}{C_{f}}+{\left(\frac{C_{p}}{C_{f}}\right)}^{2}\right)\left(\alpha -\arctan\alpha \right)\right]$$

where $f_{BW}$ denotes the circuit bandwidth and $C_{p}$ represents the parasitic capacitance. Let $\alpha =2\pi f_{BW}R_{f}C_{f}$ be defined to simplify the formula, facilitating subsequent analysis.

#### 3.1.2. Operational Amplifier Input Current Noise

The input current noise of the operational amplifier is $i_{a}$. The transfer function of this current noise to the output in the readout circuit, denoted as ${NTF}_{i}$, is given by (17).

(17) $${NTF}_{i}=\frac{R_{f}}{1+R_{f}C_{f}s}.$$

The equivalent output noise of the operational amplifier input current noise PSD is (18), and the corresponding mean-square noise voltage is (19).

(18) $$e_{i}^{2}={\left|{NTF}_{i}\right|}^{2}i_{a}^{2}.$$

(19) $$V_{n,i}^{2}=i_{a}^{2}\frac{R_{f}^{2}}{2\pi R_{f}C_{f}}\arctan\alpha$$

#### 3.1.3. Resistor Thermal Noise

The resistor thermal noise is affected by temperature, resistance value, and circuit bandwidth [19]. The resistor thermal noise is equivalent to the operational amplifier input current noise $i_{r}$.

(20) $$i_{r}=\sqrt{\frac{4KT}{R_{f}}},$$

where *K* is the Boltzmann constant, and *T* is the Kelvin temperature.

The operational amplifier output noise corresponding to the resistor thermal noise PSD is (21).

(21) $$e_{r}^{2}=\frac{4KTR_{f}}{{\left|1+R_{f}C_{f}s\right|}^{2}}.$$

Similarly, the output mean-square noise voltage generated by resistor thermal noise can be expressed as (22).

(22) $$V_{n,r}^{2}=\frac{2KT}{\pi C_{f}}\arctan\alpha .$$

### 3.2. Gyroscope Detection Limit and Output Noise

As the core detection unit of the HRG control system, the readout circuit demodulates the angular velocity signal by detecting the mechanical displacement changes of the resonator [20]. Its precision directly determines the detection limit of the control system for mechanical displacement changes. Quantitative analysis of the readout circuit output noise can provide a theoretical basis for evaluating the system’s detection limit. By equating the readout circuit output noise to the mechanical displacement noise of the resonator, i.e., equivalent displacement noise $e_{x}$, the minimum detectable displacement level of the system can be directly characterized, as expressed in (23).

(23) $$e_{x}=\frac{e_{n}}{k},$$

where $e_{n}$ represents the readout circuit output noise, and *k* represents the readout circuit gain coefficient, which characterizes the conversion relationship between the resonator displacement and the readout circuit output signal.

Since the three noise sources are mutually independent, their contributions can be summed linearly to obtain the total output noise of the readout circuit. Integration of the output noise PSD over the circuit bandwidth produces the mean-square noise voltage. Taking the square root of the mean-square noise voltage then yields the Root Mean Square (RMS) noise voltage. As shown in (24), the output noise of the transimpedance amplifier exhibits an RMS value and circuit bandwidth determined solely by internal components, independent of signal bandwidth.

(24) $$V_{n\_TIA}=\sqrt{\frac{R_{f}}{8C_{f}}i_{a}^{2}+\frac{KT}{2C_{f}}+\frac{v_{a}^{2}}{2\pi R_{f}C_{f}}\left[1+\left(2\frac{C_{p}}{C_{f}}+{\left(\frac{C_{p}}{C_{f}}\right)}^{2}\right)\left(1-\frac{\pi }{4}\right)\right]}.$$

The circuit bandwidth of the CSA is determined by the signal bandwidth, and its cutoff frequency is greater than the cutoff frequency of the RC parallel circuit in the feedback network. Based on these characteristics, the RMS noise voltage expression of the CSA is simplified, and the result is (25).

(25) $$V_{n\_CSA}=\sqrt{\frac{R_{f}}{4C_{f}}i_{a}^{2}+\frac{KT}{C_{f}}+\frac{v_{a}^{2}}{2\pi R_{f}C_{f}}\left[\alpha_{CSA}+\left(2\frac{C_{p}}{C_{f}}+{\left(\frac{C_{p}}{C_{f}}\right)}^{2}\right)\left(\alpha_{CSA}-\frac{\pi }{2}\right)\right]},$$

where $\alpha_{CSA}=2\pi f_{BW\_CSA}R_{f}C_{f}$, $f_{BW\_CSA}$ represents the signal bandwidth.

In addition, the operational amplifier voltage noise characteristics of the CSA exhibit significant frequency dependence, while the PSD of the operational amplifier current noise and resistor thermal noise are not affected by the signal bandwidth. To further reveal the contribution weights and their changing patterns of these three noise sources to the output noise of the CSA and TIA at different frequencies, modeling, simulation, and quantitative analysis will be conducted in the next section, combined with specific circuit parameters.

## 4. Simulation Analysis Experimental

### 4.1. Noise Characteristics Simulation Analysis

To analyze the noise performance differences between the TIA and the CSA, this study analyzes the noise characteristics based on the noise model established in Section 3, using the simulation platform. The input voltage and current noise parameters of the operational amplifier AD8066,which is manufactured by ADI in the Omaha, NE, USA, used in the simulation are derived from its datasheet. This chip is selected due to its extremely low input current noise, making it highly suitable for readout circuits. Table 1 presents the main parameters used in the simulation. Here, $R_{f\_TIA}$, $C_{f\_TIA}$, $R_{f\_CSA}$, and $C_{f\_CSA}$ denote the feedback network parameters of the CSA and TIA, respectively.

**Figure 6:**
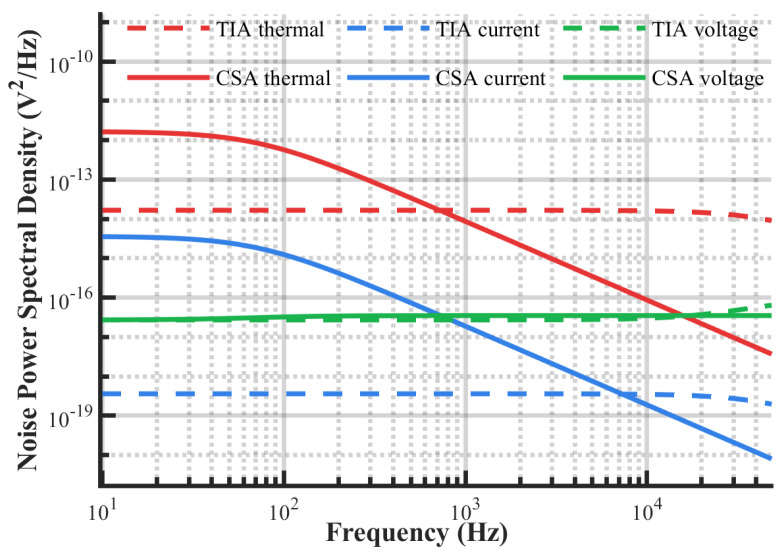
Readout circuit noise frequency characteristics.

A noise simulation model of the TIA and CSA was constructed based on the simulation platform to analyze the contribution mechanisms of each noise source in the output noise of the readout circuit, focusing on operational amplifier input voltage noise, input current noise, and resistor thermal noise. The comparison of the frequency characteristics of the two topologies is shown in Figure 6. The results demonstrate that, compared with the TIA, the CSA exhibits superior capability in suppressing both the operational amplifier’s input current noise and resistor thermal noise in the high-frequency band. As the frequency increases, the input voltage noise gradually becomes dominant. In comparison, the voltage noise, current noise, and thermal noise frequency characteristics of the TIA are relatively stable, and its noise PSD does not change with frequency within the circuit bandwidth.

**Figure 7:**
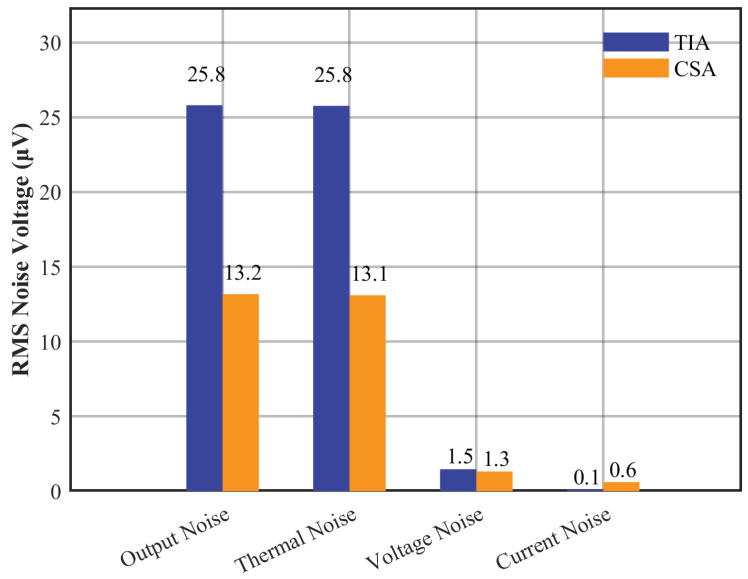
Readout circuit noise contribution mechanism.

The simulation results in Figure 7 show that the TIA output noise reaches 25.8 $\mu V_{\mathrm{rms}}$, with resistor thermal noise accounting for 99.8% of the total output noise, which verifies the dominant role of feedback resistor thermal noise. The CSA, by changing the noise transmission path, effectively suppresses the output noise, reducing it to 13.2 $\mu V_{\mathrm{rms}}$, a reduction of 48.8%. Despite the CSA’s operational amplifier current noise being approximately 6 times higher than the TIA’s, this component contributes to less than 5% of the CSA’s total output noise.

### 4.2. Noise Measurement and Results Verification

**Figure 8:**
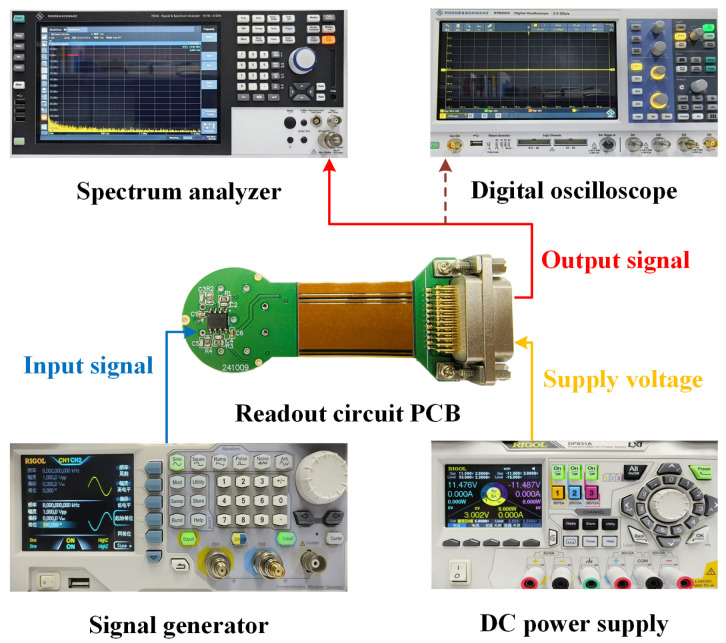
Illustration of the experimental setup of the noise measurement.

The hardware architecture of the experimental system is illustrated in Figure 8. The circuit is powered by a programmable DC power supply. The input signal is generated by a signal generator and connected to the readout circuit PCB through an input capacitor to simulate the gyroscope detection signal. After building the system, a digital oscilloscope was used to preliminarily verify the circuit function. After confirming that the function is normal, noise measurement is performed. During noise measurement, the signal input terminal is grounded to eliminate external noise interference. A spectrum analyzer measures the output noise PSD over the frequency range of 10 Hz to 50 kHz [21].

For the noise measurements, it is important to note that the output noise PSD captured by the spectrum analyzer uses a linear frequency scale on the horizontal axis. This approach ensures uniform resolution across all frequency points, enabling precise identification of anomalies at specific frequencies.

The operational amplifier uses the AD8066 low-noise dual-channel chip, with channel A configured as a TIA, with a feedback resistance of 1 MΩ and a feedback capacitance of 3.3 pF. Channel B is configured as a CSA, with a feedback resistance of 100 MΩ and a feedback capacitance of 20 pF. The circuit gain is set to 10 V/V. The dual-channel co-chip design effectively eliminates the interference of device differences on the experiment, ensuring an accurate characterization of the performance differences between the two topological structures.

**Figure 9:**
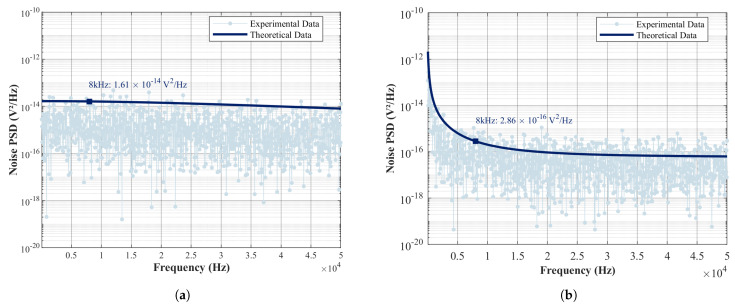
Readout circuit test results. (**a**) TIA noise data. (**b**) CSA noise data.

The test results are shown in Figure 9, where the output noise PSD of the CSA and the TIA exhibit significant differences in different frequency bands. In the low-frequency band, the noise PSD of the CSA is higher than that of the TIA, but near the resonant frequency, the noise PSD $2.86\times 10^{-16}\;V^{2}/\mathrm{Hz}$ of the CSA is reduced by approximately two orders of magnitude compared to the noise PSD $1.61\times 10^{-14}\;V^{2}/\mathrm{Hz}$ of the TIA, indicating that the CSA exhibits significantly better high-frequency noise suppression capability than the TIA. The slight deviation between theoretical and experimental data observed in the experiment is mainly due to external interference in the test environment and the uncertainty of the noise itself. Further combining the obtained displacement–voltage gain coefficient of $1\times 10^{6}\;V/m$, the equivalent mechanical displacement noise of the CSA near the resonant frequency can be calculated as $1.69\times 10^{-14}\;m/\sqrt{\mathrm{Hz}}$, representing an 86.7% reduction compared to $1.27\times 10^{-13}\;m/\sqrt{\mathrm{Hz}}$ of the TIA, indicating that the CSA is more suitable for use in HRG readout circuits.

## 5. Conclusions

This paper addresses the noise issues in the readout circuit of the HRG. According to the HRG detection principle, the circuit characteristics of the TIA and the CSA are comparatively analyzed. Noise models for both TIA and CSA are established, and their noise contribution mechanisms along with frequency characteristics are theoretically analyzed. Through noise model simulations, the primary noise source is identified as resistor thermal noise. The output noise of the TIA is 25.8 $\mu V_{\mathrm{rms}}$, with the feedback resistor’s thermal noise accounting for 99.8%. Conversely, the CSA feedback network is dominated by capacitance, which effectively suppresses the resistor’s thermal noise at high frequencies, reducing its output noise to 13.2 $\mu V_{\mathrm{rms}}$, a 48.8% reduction compared to the TIA. Experimental tests confirm consistency between measured and simulated noise, validating the accuracy of the theoretical model. Near the resonant frequency, the equivalent displacement noise of the CSA is $1.69\times 10^{-14}\;m/\sqrt{\mathrm{Hz}}$, a reduction of 86.7% compared to the TIA’s $1.27\times 10^{-13}\;m/\sqrt{\mathrm{Hz}}$, demonstrating the CSA is more suitable for use in HRG readout circuits. These findings provide theoretical guidance for selecting topologies and designing parameters in high-precision HRG readout circuits, thereby enhancing gyroscope control system stability and detection accuracy.

## Figures and Tables

**Table 1:** Main parameters of the simulation noise model.

Parameter	Setting Value	Unit
$v_{a}$	$7\times 10^{-9}$	$V/\sqrt{\mathrm{Hz}}\;$
$i_{a}$	$6\times 10^{-16}$	$A/\sqrt{\mathrm{Hz}}\;$
*K*	$1.38\times 10^{-23}$	J/K
*T*	300	K
$C_{p}$	$2\times 10^{-12}$	F
$R_{f\_TIA}$	$1\times 10^{6}$	Ω
$C_{f\_TIA}$	$3.3\times 10^{-12}$	F
$R_{f\_CSA}$	$1\times 10^{8}$	Ω
$C_{f\_CSA}$	$2\times 10^{-11}$	F
$f_{BW}$	$5\times 10^{4}$	Hz

## Data Availability

The data used to support the findings of this study are available from the corresponding author upon request.
